# Microbiological Profile of Corneal Ulcers at a Tertiary Referral Center

**Published:** 2019

**Authors:** Mohammad ZARE, Peyman Mohammadi TORBATI, Fahimeh ASADI-AMOLI, Mohammadreza TALEBNEJAD, Maryam PARVIZI, Zahra NASIRI, Reza GHAREBAGHI, Fatemeh HEIDARY

**Affiliations:** 1 Ophthalmic Research Center, Department of Ophthalmology, Labbafinejad Medical Center, Shahid Beheshti University of Medical Sciences, Tehran, Iran; 2 Department of Pathology, Labbafinejad Hospital, Shahid Beheshti University of Medical Sciences, Tehran, Iran; 3 Department of Pathology, Tehran University of Medical Sciences, Tehran, Iran; 4 Department of Ophthalmology, Poostchi Ophthalmology Research Center, Shiraz University of Medical Sciences, Shiraz, Iran; 5 Department of Pathology, Mofid children's Hospital, Shahid Beheshti University of Medical Sciences, Tehran, Iran; 6 Faculty of Mathematical Sciences, Tarbiat Modares University, Tehran, Iran; 7 International Virtual Ophthalmic Research Center, Tehran, Iran; 8 Immunoregulation Research Center, Shahed University, Tehran, Iran

**Keywords:** Microbiological Profile, Antibiotic Susceptibility, Corneal Ulcer, Tertiary referral center

## Abstract

The aim of this study was to describe patient demographics, microbiological profile, and antibiotic susceptibility of corneal ulcer at a tertiary referral center to improve and optimize diagnosis and treatment of this potentially blinding entity and to reduce antibiotic misuse. Detailed external and slit-lamp bio-microscopic examination of 123 consecutive patients with suspected corneal ulcer was performed at an ophthalmology clinic. Corneal scraping was carried out under slit-lamp bio-microscopy. The obtained material was inoculated on culture media and smeared on a slide for Gram's staining for morphological identification of bacteria and fungus. For samples that developed colony in culture media, antibiotic susceptibility testing was performed. In a significant percentage of patients (72%) neither bacterial agents nor fungi were the cause of corneal ulcer. Of the 34 culture-proven corneal ulcers, in 79% of the cases, bacteria were detected while in 21% of cases, fungi were found. Of the 27 bacterial corneal ulcers, the majority were (67%) caused by Gram-positive bacteria, of which 50% were *Streptococcus pneumoniae*, and in the Gram-negative bacterial corneal ulcers, most of the cases (44%) were caused by *Pseudomonas aeruginosa*. In the antibiotic susceptibility report, *Streptococcus pneumoniae, Staphylococcus aureus, Pseudomonas aeruginosa*, and *Escherichia coli* were resistant to Cotrimoxazole (TS), *Streptococcus pneumoniae* to Erythromycin (E), *Staphylococcus aureus* to Peniciline (PG), *Pseudomonas aeruginosa* to Ceftriaxone (CRO) and Nitrofurantoin (NI), and finally, *Escherichia coli* to Gentamicin (GM). In conclusion, in a significant number of the patients neither bacterial agents nor fungi were offending microorganisms and bacteria were the most common agent of microbiological corneal ulcer, found in 79% of culture-proven corneal ulcers, followed by fungus, found in 21% of culture-proven corneal ulcers.

## INTRODUCTION

A “silent epidemic” is a term that refers to corneal ulceration in developing countries and its incidence is approximately ten times that of the United States [1], which annually imposes a heavy financial burden on the health-care system, even in developed countries [2]. Even though the most common offending agent is Herpes Simplex Virus type 1, other common infectious agents in this potentially blinding condition are bacteria, including *Streptococcus pneumonia, Staphylococcus aureus, coagulase-negative staphylococci, *and* Pseudomonas aeruginosa*; fungus including *Candida albicans, Aspergillus flavus, Fusarium solani, Penicillium species *and* Aspergillus fumigates* as well as parasites [3, 4]. Environmental and geographical factors can influence the pattern of infection and there are numerous differences in the profile of corneal ulcers in various regions [5].

Although effective treatment with fortified antibiotics based on the results of corneal smear and culture is necessary, in the management of this sight-threatening condition, intensive treatment with several topical broad-spectrum fortified antibiotics is used while culture results are pending [6]. However, the emergence of a wide variety of antibiotic resistance has been reported in the recent decades, such as fluoroquinolones resistance in case of culture-proven corneal ulcer with prior use of this antibiotic or moxifloxacin resistance in corneal ulcer caused by *Pseudomonas aeruginosa* [6-10]. These findings highlight the necessity of conducting epidemiological studies to examine antibiotic susceptibility in different geographical areas so that a cost-effective approach can be designed for the management of this infective condition. Meanwhile, such studies will help the selection of effective antibiotics for optimal treatment of corneal ulcer in that specific geographical area based on previous studies conducted in this area.

The aim of this study was to describe patient demographics, microbiological profile, and antibiotic susceptibility of corneal ulcer at a tertiary referral center to improve and optimize diagnosis and treatment of this potentially blinding entity and also to reduce antibiotic misuse as well as antibiotic resistance of the microbes.

## METHODS

This retrospective cross-sectional study was conducted between 23^rd^ of September 2017 to 23^rd^ of September 2018 at the pathology laboratory of Shaheed Labbafinejad Hospital, Shaheed Beheshti University of Medical Sciences, Tehran, Iran. The study obtained ethical approval from the Ethical Committee of the Shahid Beheshti University of Medical Sciences (the research ethic's code is: IR.SBMU.RETECH.REC.1397.863). Following the Declaration of Helsinki, the samples were collected as a routine practice of ophthalmology clinic for suspected infectious corneal ulcers.

Detailed external and slit-lamp bio-microscopic examination of 123 consecutive patients with suspected corneal ulcer was performed by a resident of ophthalmology. Corneal scraping was carried out under slit-lamp magnification, following instillation of one drop of 0.5% tetracaine hydrochloride (Anestocaine, Sinadaru, Tehran, Iran) in an aseptic condition, from the leading edge and base of the ulcer, using a sterile No. 15 Bard-Parker.

The obtained material was inoculated on culture media, including MacConkey agar (Pronadisa Lab. Conda, Madrid, Spain), Blood Agar Pronadisa medium (Pronadisa Lab. Conda, Madrid, Spain), chocolate agar (Pronadisa Lab. Conda, Madrid, Spain), and Sabouraud dextrose agar (Pronadisa Lab. Conda, Madrid, Spain) for culture; the methods were based on instructions of the manual of American Society for Microbiology [11] and smear on the slide for Gram's stain for morphological identification of bacteria and fungus.

For samples that developed colonies in culture media, antibiotic susceptibility testing was performed using the following Mast Discs (Mast Group, Merseyside, UK): cefazolin (30 μg) – CZ30; streptomycin (10 μg) – S10; penicillin G (10 μg) – PG10; novobiocin (5 μg) – NO5; Cefepime (30 μg) – CPM; erythromycin (15 μg) - E15; nitrofurantoin (200 μg) – NI200; cefoxitin (30 μg) – FOX30; cotrimoxazole (Trimethoprim/sulfamethoxazole 1.25/23.75 μg) - TS 25C; Amikacin (30 μg) - AK30; Ceftazidime (5 μg) - CAZ5; ceftriaxone (30 μg) – CRO30; Meropenem (10 μg) - MEM10; Ampicillin (25 μg) - AP25; Imipenem (10 μg) - IMI10; vancomycin (30 μg) – VA30; gentamicin (10 μg), – GM10; and ciprofloxacin (1 μg) - CIP1C. An antibiotic disc was selected based on Gram staining of a developed colony in culture media and specific features of colonies showing the potential microorganism. In the microbiological report of this test, the sensitivity or resistance to the antibiotic was reported for each sample. 

The obtained data was entered in Microsoft Office Excel, 2016 and Statistical Package for Social Science software (ver. 22.0, SPSS, Inc., Chicago, IL) was used for analysis of data. Statistical analysis included both descriptive and analytical statistics. Frequency, percentage, mean, and standard deviation (SD) for data were reported. In analytical statistics, P <0.05 was considered significant.

## RESULTS

Overall, the mean ± SD age of 123 enrolled patients in this study was 53.88±16.76 years old, of which 68 were men and 55 were women, with a mean ± SD age of 58.21±15.03 years and 48.53±17.37 years, respectively, where women were significantly younger than men (P < 0.001).

In total, among 89 (72%) cases of corneal ulcers, no microorganisms in Gram stain and culture media were detected, and in the remaining 34 patients, who had culture-proven infectious corneal ulcer (Table 1), the following microorganisms were identified in order of frequency: *Streptococcus pneumoniae* in nine patients (six men and three women), *Staphylococcus aureus* in seven patients (seven women), *Pseudomonas aeruginosa *and* Candida albicans*; each in four patients (*Pseudomonas aeruginosa*; three men and one woman and *Candida albicans*; one man and three women), *Escherichia coli* in three patients (two men and one woman), *Aspergillus flavus* in two patients (two men), and *Acinetobacter spp, Alternaria spp, Klebsiella pneumoniae*, alpha-hemolytic *Streptococcus *and* Streptococcus agalactiae,* each detected in one case (one woman, one man, one woman, one woman, and one man, respectively). According to the Gram staining results, out of 123 smears, eighteen Gram-positive and nine Gram-negative bacteria were detected. In order of frequency, out of 34 infectious corneal ulcers, 18 (53%) were caused by Gram-positive bacteria, nine (26%) by Gram- negative bacteria, and seven (21%) by fungus. 

The results of antibiotic susceptibility testing for 27 bacterial keratitis out of 34 infectious corneal ulcers is shown in Table 2. These data were collected from the microbiology laboratory notebook, and no extra information other than the name of resistant or sensitive antibiotic was found.

**Table 1:** Microbiological Results for Culture-proven Infectious Corneal Ulcers (n = 34)

Organism	Number
Gram –positive bacteria	
*** Streptococcus pneumoniae***	9
*** Staphylococcus aureus***	7
*** alpha-hemolytic streptococcus***	1
*** Streptococcus agalactiae***	1
Total	**18**
Gram –negative bacteria	
*** Pseudomonas aeruginosa***	4
*** Escherichia coli***	3
*** Klebsiella pneumoniae***	1
*** Acinetobacter spp***	1
Total	**9**
Fungus	
*** Candida albicans***	4
*** Aspergillus flavus***	2
*** Alternaria spp***	1
Total	**7**

**Table 2:** Result of Antibiotic Susceptibility testing for 27 Culture-Proven Bacterial Corneal Ulcers

Organism	Resistant	Sensitive
*Streptococcus pneumoniae*	TS, E	VA, CRO, MEM, S
*Staphylococcus aureus*	TS, PG	VA, CZ, NO, FOX
*Pseudomonas aeruginosa*	TS, CRO, NI	AK, GM, CIP, IMI,CPM, CAZ
*Escherichia coli*	TS, GM	CRO, CIP, CAZ, MEM, IMI, AK
*Acinetobacter spp*	-	AK, CAZ, CRO, GM, IMI, MEM
*Klebsiella pneumoniae*	-	AK, CAZ, CRO, CIP, GM, IMI
*Alpha-hemolytic streptococcus*	-	VA, E, CRO, MEM
*Streptococcus agalactiae*	-	AP, IMI, VA, GM, CIP

PG: Peniciline; S: Streptomycin; CZ: Cefazolin; NO: Novobiocin; CPM: Cefepime; E: Erythromycin; NI: Nitrofurantoin; FOX: Cefoxitin; TS: Cotrimoxazole; AK: Amikacin; CAZ: Ceftazidime; CRO: Ceftriaxone; MEM: Meropenem; AP: Ampicillin; IMI: Imipenem; VA: Vancomycin; GM: Gentamicin; CIP: Ciprofloxacin.

## DISCUSSION

In the present study, the majority of patients were male, who were significantly older than females, and in a significant percentage of patients (72%), neither bacterial agents nor fungi were the cause of corneal ulcer. Of the 34 culture-proven corneal ulcers, offending microorganisms in 79% of the cases were bacteria and in 21% of the case, these were fungi. Of the 27 bacterial corneal ulcers, the majority (67%) were caused by Gram-positive bacteria, of which 50% were *Streptococcus pneumoniae,* and in the Gram-negative bacterial corneal ulcers, most of the cases (44%) were caused by *Pseudomonas aeruginosa*. Among seven cases of fungal corneal ulcers, *Candida albicans* was detected in four patients (57%), *Aspergillus flavus* in two patients (29%) and *Alternaria spp* in one patient (14%) as the offending fungus. In antibiotic susceptibility report, *Streptococcus pneumoniae, Staphylococcus aureus, Pseudomonas aeruginosa*, and *Escherichia coli* were resistant to TS, *Streptococcus pneumoniae* to E, *Staphylococcus aureus* to PG, *Pseudomonas aeruginosa* to CRO and NI, and finally, *Escherichia coli* to GM.

In the current study, culture proven microbial corneal ulcers were detected in 28% of corneal scrapings. This was lower than reports from Nepal [12], Ghana [13], South India [14], and Bangladesh [15], which was 45.5%, 57.3%, 70.6%, and 81.7%, respectively. 

In the present study, bacteria were the most common agents of microbiological corneal ulcer in 79% of the culture-proven corneal ulcers and 22% of all patients with clinical diagnosis of corneal ulcer, followed by fungus in 21% of culture-proven corneal ulcers and 6% of all clinical diagnoses of corneal ulcer. In studies by Tewari et. al [16] and Suwal et. al. [4] similar results were found. In Tewari’s study, bacterial agents caused 65% of the culture-proven corneal ulcers, and the fungus was the offending microorganism in 35% of the cases, and similar to the present study, Gram-positive cocci were the most common bacteria, and among the Gram-negative bacteria, *Pseudomonas aeruginosa* was the most common. Unlike the present study, however, among the gram-positive bacteria, *Staphylococcus aureus* and among fungal causes, *Aspergillus spp*. followed by *Fusarium* spp. were the most common, which could be due to differences in risk factors and geographical distribution among the two studies. However, because of the retrospective nature of the current study design, the risk factors were not recorded, thus, the causes of these differences are not certain. In Suwal’s study, bacteria were detected in the majority of the culture-proven corneal ulcers, amongst which *Streptococcus pneumoniae* was the most common bacteria, as in the present study. Although the fungus was the second common agent of microbiological corneal ulcers, similar to the present study, the most common fungi were *Fusarium* species followed by *Aspergillus flavus*, which differs from the findings of the current study; the most likely justification for this difference is that mentioned previously.

The emergence of antibiotic resistance is a major public health issue and the distribution of microorganisms causing corneal ulcers, especially those which are resistant to antibiotics, varies according to time, geographical location, and hospital [17]. In the study of Mohammadpour et al. [18] on antibiotic susceptibility patterns in pseudomonas corneal ulcers, this microbe was susceptible to CAZ, CIP and AK, as was in the current study. Although the current study was conducted at a different tertiary referral hospital, the study city and the population studied were the same in both studies, which could explain the similarity of the results. In another study conducted in Iran [19] at a tertiary referral center, all *Pseudomonas aeruginosa*, such as the current study, were susceptible to CAZ and AK. In that study, only about 7% were resistant to CIP, whereas, in the present study, all *Pseudomonas aeruginosa* were resistant to TS, CRO, and NI. Also, their study results showed that *Streptococcus pneumoniae* were resistant to AK in 2.6% and in all cases sensitive to CZ, CAZ, cefixime, and cephalothin. However, in the current study, all *Streptococcus pneumoniae* were resistant to TS and E and sensitive to VA, CRO, MEM, and S.

The study design, recruitment of subjects, and its short duration can be cited as the limitations of this study. Due to the retrospective nature of this study, important information, which could be considered as possible risk factors of corneal ulcer, such as socioeconomic status of patients, history of contact lens wear, and occupational exposure, besides consequent complications of corneal ulcer was not available in the patients’ records. Likewise, the choice of antibiotic discs to detect antibiotic sensitivity was out of the preference of the authors of this paper, and the authors only recorded the information in the microbiology laboratory notebook. Therefore, in order to make thoughtful decisions to reduce the risk of corneal ulcer and manage this potentially blinding condition, the need for stronger study design and a longer follow-up period is considered necessary besides examining predisposing factors, such as occupational exposure, history of contact lens wear, and socioeconomic level of patients and vigilance selection of antibiotic discs for antibiotic sensitivity testing.

## CONCLUSIONS

Overall, in a significant percentage of patients neither bacterial agents nor fungi were offending microorganisms and bacteria were the most common agent of microbiological corneal ulcer in 79% of the culture-proven corneal ulcers, followed by fungus in 21% of the culture-proven corneal ulcers. In antibiotic susceptibility report, *Streptococcus pneumoniae,* *Staphylococcus aureus, Pseudomonas aeruginosa, *and* Escherichia coli* were resistant to TS, *Streptococcus pneumoniae* to E, *Staphylococcus aureus* to PG, *Pseudomonas aeruginosa* to CRO and NI, and finally*, Escherichia coli* to GM.

## References

[1] Whitcher JP, Srinivasan M (1997). Corneal ulceration in the developing world--a silent epidemic. Br J Ophthalmol.

[2] Collier SA, Gronostaj MP, MacGurn AK, Cope JR, Awsumb KL, Yoder JS (2014). Estimated burden of keratitis--United States, 2010. MMWR Morb Mortal Wkly Rep.

[3] Green M, Apel A, Stapleton F (2008). Risk factors and causative organisms in microbial keratitis. Cornea.

[4] Suwal S, Bhandari D, Thapa P, Shrestha MK, Amatya J (2016). Microbiological profile of corneal ulcer cases diagnosed in a tertiary care ophthalmological institute in Nepal. BMC Ophthalmol.

[5] Schaefer F, Bruttin O, Zografos L, Guex-Crosier Y (2001). Bacterial keratitis: a prospective clinical and microbiological study. Br J Ophthalmol.

[6] Alexandrakis G, Alfonso EC, Miller D (2000). Shifting trends in bacterial keratitis in south Florida and emerging resistance to fluoroquinolones. Ophthalmol.

[7] Zhang C, Liang Y, Deng S, Wang Z, Li R, Sun X (2008). Distribution of bacterial keratitis and emerging resistance to antibiotics in China from 2001 to 2004. Clin Ophthalmol.

[8] Ray KJ, Prajna L, Srinivasan M, Geetha M, Karpagam R, Glidden D (2013). Fluoroquinolone treatment and susceptibility of isolates from bacterial keratitis. JAMA Ophthalmol.

[9] Oldenburg CE, Lalitha P, Srinivasan M, Rajaraman R, Ravindran M, Mascarenhas J (2013). Emerging moxifloxacin resistance in Pseudomonas aeruginosa keratitis isolates in South India. Ophthalmic Epidemiol.

[10] Fintelmann RE, Hoskins EN, Lietman TM, Keenan JD, Gaynor BD, Cevallos V (2011). Topical fluoroquinolone use as a risk factor for in vitro fluoroquinolone resistance in ocular cultures. Arch Ophthalmol.

[11] Murray Patrick R, Baron Eleen J (2007). Manual of Clinical Microbiology.

[12] Lavaju P, Arya SK, Khanal B, Amatya R, Patel S (2009). Demograhic pattern, clinical features and treatment outcome of patients with infective keratitis in the eastern region of Nepal. Nepal J Ophthalmol.

[13] Leck AK, Thomas PA, Hagan M, Kaliamurthy J, Ackuaku E, John M (2002). Aetiology of suppurative corneal ulcers in Ghana and south India, and epidemiology of fungal keratitis. Br J Ophthalmol.

[14] Bharathi MJ, Ramakrishnan R, Vasu S, Meenakshi R, Palaniappan R (2003). Epidemiological characteristics and laboratory diagnosis of fungal keratitis A three-year study. Indian J Ophthalmol.

[15] Dunlop AA, Wright ED, Howlader SA, Nazrul I, Husain R, McClellan K (1994). Suppurative corneal ulceration in Bangladesh A study of 142 cases examining the microbiological diagnosis, clinical and epidemiological features of bacterial and fungal keratitis. Aust N Z J Ophthalmol.

[16] Tewari A, Sood N, Vegad MM, Mehta DC (2012). Epidemiological and microbiological profile of infective keratitis in Ahmedabad. Indian J Ophthalmol.

[17] Chalita MR, Hofling-Lima AL, Paranhos A Jr, Schor P, Belfort R, Jr (2004). Shifting trends in in vitro antibiotic susceptibilities for common ocular isolates during a period of 15 years. Am J Ophthalmol.

[18] Mohammadpour M, Mohajernezhadfard Z, Khodabande A, Vahedi P (2011). Antibiotic susceptibility patterns of pseudomonas corneal ulcers in contact lens wearers. Middle East Afr J Ophthalmol.

[19] Rahimi F, Hashemian MN, Khosravi A, Moradi G, Bamdad S (2015). Bacterial keratitis in a tertiary eye centre in Iran: a retrospective study. Middle East Afr J Ophthalmol.

